# Short-term outcome following surgery for rare brain tumor entities in adults: a Swedish nation-wide registry-based study and comparison with SEER database

**DOI:** 10.1007/s11060-020-03490-z

**Published:** 2020-05-19

**Authors:** Jiri Bartek, Sanjay Dhawan, Erik Thurin, Ali Alattar, Sasha Gulati, Bertil Rydenhag, Roger Henriksson, Clark C. Chen, Asgeir Store Jakola

**Affiliations:** 1 Department of Neurosurgery, Karolinska University Hospital, Stockholm, Sweden; 2 Department of Clinical Neuroscience and Department of Medicine, Karolinska Institutet, Stockholm, Sweden; 3 Department of Neurosurgery, Copenhagen University Hospital Rigshospitalet, Copenhagen, Denmark; 4 Department of Neurosurgery, University of Minnesota, Minneapolis, MN USA; 5 Institute of Neuroscience and Physiology, University of Gothenburg, Sahlgrenska Academy, Gothenburg, Sweden; 6 Department of Neurosurgery, University of Pittsburgh Medical Center, Pittsburgh, PA USA; 7 Department of Neurosurgery, St. Olav University Hospital, Trondheim, Norway; 8 Department of Radiation Sciences, University of Umeå, S-901 85 Umeå, Sweden; 9 Department of Neurosurgery, Sahlgrenska University Hospital, Gothenburg, Sweden

**Keywords:** Neurosurgery, Outcome, Adults, Morbidity, Complications, Registry, Ganglioglioma, Ependymoma, Subependymoma, Pilocytic astrocytoma, Primitive neuroectodermal tumor

## Abstract

**Objective:**

To investigate outcomes after surgery for rare brain tumors using the Swedish Brain Tumor Registry (SBTR).

**Methods:**

This is a nationwide study of patient in the SBTR, validated in the Surveillance, Epidemiology, and End Results (SEER) registries. We included all adults diagnosed 2009–2015 with a rare brain tumor entity (n = 216), defined as ependymoma (EP, n = 64), subependymoma (SUBEP, n = 21), ganglioglioma (GGL, n = 54), pilocytic astrocytoma (PA, n = 56) and primitive neuroectodermal tumor (PNET, n = 21). We analyzed symptomatology, tumor characteristics and outcomes.

**Results:**

Mean age was 38.3 ± 17.2 years in GGL, 36.2 ± 16.9 in PA, 37.0 ± 19.1 in PNET, 51.7 ± 16.3 in EP and 49.8 ± 14.3 in SUBEP. The most common symptom was focal deficit (39.6–71.4%), and this symptom was most common in GGL patients with 64.2% of GGL presenting with seizures. Most patients had no or little restriction in activity before surgery (Performance Status 0–1), although up to 15.0% of PNET patients had a performance status of 4. Gross total resection was achieved in most (> 50%) tumor categories. Incidence of new deficits was 11.1–34.4%. In terms of postoperative complications, 0–4.8% had a hematoma of any kind, 1.9–15.6% an infection, 0–7.8% a venous thromboembolism and 3.7–10.9% experienced a complication requiring reoperation. There were 3 deaths within 30-days of surgery, and a 1-year mortality of 0–14.3%.

**Conclusion:**

We have provided benchmarks for the current symptomatology, tumor characteristics and outcomes after surgery for rare brain tumors as collected by the SBTR and validated our results in an independent registry. These results may aid in clinical decision making and advising patients.

## Introduction

Brain tumor surgery is considered a high-risk endeavor [1, 2], especially to consider in the management of rare brain tumors when the personal and even the institutional experience remains limited. Among the ‘rare brain tumors’ are entities such as ganglioglioma (GGL, < 1% of adult intracranial tumors), pilocytic astrocytoma (PA, < 1.5% of adult intracranial tumors), medulloblastoma/primitive neuroectodermal tumors (PNET, < 1.0% of adult tumors), ependymoma (EP, < 1.9% of adult tumors) and subependymoma (SUBEP, < 0.7% of adult intracranial tumors) [3]. Given the rarity of these entities in the adult population, previous reports have mainly focused on the treatment, with systematic data on surgical outcome given little attention [4]. In rare brain tumors, estimates of prognosis and the risks of surgery are likely inaccurate, and certainly less informed than estimates in common tumors. Some of these lesions also have a benign natural course of disease, making it especially important to carefully consider the risk and benefits of surgery given the longevity of this patient population [1]. Even in cases when surgery is unavoidable, the potential of short-term risk is relevant since patients and relatives can be better informed of expected clinical course and potential complications. Well informed patients are likely to cope better when deviations from the optimal post-operative course is encountered.

A limitation for the generalizability of previously few publications is that some are based on non-consecutive and non-population-based material (i.e. patients only treated in selected centers) and are mainly mixed pediatric/adult series [5–8]. To fill this knowledge gap, we conducted a Swedish nationwide register-based study in the modern era to capture short-term surgical risk profile. We then validate our results in the United States Surveillance, Epidemiology, and End Results (SEER) database.

## Materials and methods

### The Swedish brain tumor registry

The SBTR is a regionally based registry of adult (18 years or older) patients with diagnosed brain tumors that started in 1999. All regions report to SBTR; however, the level of coverage has varied between the different regions over time. For further details of the registry, see Asklund et al. [9]. The variables registered in the SBTR following surgery are described in detail in Table 1.

**Table 1:** Definitions of variables in the SBTR registry

Variable	Definition
Age	Years at time of diagnosis
Sex	Male or female
Symptoms at diagnosis	Asymptomatic (yes/no) Seizure (yes/no) Focal deficit (yes/no) “ICP related” (e.g. headache, cognition) (yes/no)
WHO performance status	0—asymptomatic 1—symptomatic but completely ambulatory 2—symptomatic, < 50% in bed during the day 3—symptomatic, > 50% in bed during the day but not bedbound Bedbound Death
Date of imaging diagnosis	dd.mm.yyyy
Location Based on coding equivalent to ICD-10 even though C71 codes not necessarily used for clinical coding	C71.1: frontal C71.2: temporal C71.3: parietal C71.4: occipital C71.5: ventricular C71.6: cerebellar C71.7: brainstem C71.8: whole brain C71.9: not specified
Laterality	Left/right/bilateral
Multifocal	Yes/no
Largest diameter of tumor	< 4 cm 4–6 cm > 6 cm
Type of surgery	Biopsy or resection
Date of surgery	dd.mm.yyyy
Tumor removal as determined by the surgeon	Biopsy (only tissue diagnosis) Partial resection Gross total resection
New or worsened focal deficit within 30 days	Yes/no
New onset seizure within 30 days	Yes/no
Any infection within 30 days	Yes/no
Any VTE within 30 days	Yes/no
Any hematoma within 30 days	Yes/no
Complication leading to reoperation within 30 days	Yes/no
Date of discharge neurosurgical department	dd.mm.yyyy
Histopathology	*SNOMED codes:* *93,831 subependymoma* *93,913, 93,923 ependymomas* *94,130 DNET* *94,211 Pilocytic astrocytoma* *94,703, 94,713 medulloblastoma* *94,920 Gangliocytoma* *94,930, 95,951 Ganglioglioma*

### Definition of cohort

Using the SBTR, we aimed to include all patients with rare brain tumor entities treated in Sweden from 2009 through 2015 to provide actuality of the current neurosurgical practice. However, in our study we have only used data from regions where the total registration for all tumor entities was 80% or more for any given year to provide population-based data. Registration rate was defined as percentage of diagnoses in the SBTR that corresponds to diagnoses reported to the compulsory National Cancer Registry.

### Surveillance, Epidemiology, and End Results (SEER) database

The SEER registry (https://seer.cancer.gov/about/) collects data on cancer incidence and patient survival rates in the United States. We used patient data from SEER database representing age at diagnosis, sex, tumor characteristics (size, laterality, location, World Health Organization (WHO) grade), extent of resection and overall survival for adult patients with medulloblastoma (including PNET), EP, SUBEP, GGL, PA. International Classification of Diseases for Oncology (ICD-O-3) morphology codes were used to define cases: medulloblastoma (including PNET) (9470–9478); EP (9391, 9394); SUBEP (9383); GGL (9505); PA (9421).

### Statistics

All analyses of SBTR data were done with SPSS, version 24.0 (Chicago, IL, USA). Statistical significance level was set to p < 0.05. All tests were two-sided. Central tendencies were presented as means ± SD, or median and interquartile range if skewed. Categorical data were analyzed with Pearson´s chi-square test. For survival we presented Kaplan–Meier curves. Data from the SEER database was analyzed using RStudio Version 0.99.467 (RStudio, Boston, Massachusetts, USA).

## Results

A total of 226 patients included in the SBTR were identified. Very small subgroups were excluded for further analyses, consequently gangliocytomas (5 patients) and DNET (5 patients) are not part of further analyses that consisted of 216 patients. Among these, there were 54 (25.0%) patients with GGL, 56 (25.9%) patients with PA, 21 (9.7%) patients with the WHO 2007 diagnosis of PNET, 64 (29.6%) patients with EP, and 21 (9.7%) patients with SUBEP.

### Ganglioglioma

Mean age was 38.3 ± 17.2 years and there was a slight male predominance (55.6%). The primary locations were frontal (27.8%) and temporal (35.2%). Seizures were present at initial presentation in 64.2% of patients. Only 3.7% of patients were asymptomatic upon presentation, but the majority had no restrictions in their daily activity (WHO performance status 0, 64.2%). Gross total resection was achieved in 63.0% of patients. New deficits occurred in 11.1% after surgery. Two patients (3.7%) had a reoperation due to complication. There were no cases of postoperative hematoma, venous thromboembolism or deaths within 30 days of surgery. For further details see Tables 2 and 3.

**Table 2:** SBTR patients baseline characteristics

	GGL (n = 54)	PA (n = 56)	PNET (n = 21)	EP (n = 64)	SUBEP (n = 21)
Age, mean (SD) Missing, n = 1	38.3 (17.2)	36.2 (16.9)	37.0 (19.1)	51.7 (16.3)	49.8 (14.3)
Female, n (%)	24 (44.4)	26 (46.4)	7 (33.3)	32 (50.0)	4 (19.0)
Preop MRI, n (%)	52 (96.3)	54 (96.4)	21 (100)	61 (95.3)	19 (90.5)
Tumor size, n/N (%)					
< 4 cm	22 (50.0)	34 (66.7)	9 (50.0)	38 (69.1)	16 (84.2)
4–6 cm	16 (36.4)	11 (21.6)	8 (44.4)	15 (27.3)	3 (15.8)
> 6 cm Missing, n = 29	6 (13.6)	6 (11.8)	1 (5.6)	2 (3.6)	0
Multifocal, n (%) Missing, n = 1	3 (5.7)	4 (7.1)	5 (23.8)	5 (7.8)	1 (4.8)
Laterality					
Right, n (%)	28 (58.3)	22 (53.7)	6 (40.0)	18 (81.8)	7 (70.0)
Left, n (%)	20 (41.7)	19 (46.3)	7 (46.7)	3 (13.6)	3 (30.0)
Bilateral, n (%) Missing, n = 80	0	0	2 (13.3)	1 (4.5)	0
Location, n (%)					
Frontal	15 (27.8)	9 (16.1)	1 (4.8)	1 (1.6)	0
Temporal	19 (35.2)	3 (5.4)	2 (9.5)	2 (3.1)	0
Parietal	4 (7.4)	4 (7.1)	0	1 (1.6)	0
Occipital	4 (7.4)	4 (7.1)	0	2 (3.1)	0
Ventricle	2 (3.7)	0	1 (4.8)	5 (7.8)	6 (28.6)
Cerebellum	2 (3.7)	21 (37.5)	15 (71.4)	7 (10.9)	3 (14.3)
Brain stem/4th ventricle	0	6 (10.7)	1 (4.8)	36 (56.3)	11 (52.4)
Corpus callosum	1 (1.9)	0	0	1 (1.6)	0
Unspecified	7 (13.0)	9 (16.1)	0	9 (14.0)	1 (4.8)
WHO grade, n (%)					
I	48 (88.8)	56 (100)	0	0	21 (100)
II	0	0	0	60 (93.7)	0
III	6 (11.1)	0	0	4 (6.3)	0
VI	0	0	21 (100)	0	0
Asymptomatic, n (%)	2 (3.7)	4 (7.1)	0	5 (7.8)	5 (23.8)
Focal deficit, n (%) missing, n = 8	21 (39.6)	27 (50.9)	15 (71.4)	26 (42.6)	8 (40.0)
Seizures, n (%) Missing, n = 8	34 (64.2)	14 (26.4)	1 (4.8)	6 (9.8)	0
ICP related, n (%) Missing, n = 8	16 (30.2)	31 (58.5)	17 (81.0)	41 (67.2)	13 (65.0)
Performance status, n (%)					
0	34 (64.2)	29 (51.8)	5 (25.0)	22 (36.1)	8 (40.0)
1	12 (22.6)	14 (25.0)	6 (30.0)	20 (32.8)	10 (50.0)
2	7 (13.2)	8 (14.3)	6 (30.0)	11 (18.0)	2 (10.0)
3	0	3 (5.4)	0	7 (11.5)	0
4 Missing, n = 6	0	2 (3.6)	3 (15.0)	1 (1.6)	0
Imaging diagnosis to surgery, median, months (IQR) Missing, n = 1	2 (0–5)	0 (0–2)	0 (0–0)	0 (0–2)	1 (0–8)

**Table 3:** SBTR patients intraoperative and postoperative variables

	GGL (n = 54)	PA (n = 56)	PNET (n = 21)	EP (n = 64)	SUBEP (n = 21)
Tumor removal, n (%)					
Biopsy	6 (11.1)	5 (8.9)	2 (9.5)	1 (1.6)	0
Partial	14 (25.9)	20 (35.7)	7 (33.3)	24 (37.5)	2 (9.5)
Gross total	34 (63.0)	31 (55.4)	12 (57.1)	39 (60.9)	19 (90.5)
New deficit, n (%)	6 (11.1)	11 (19.6)	7 (33.3)	22 (34.4)	4 (19.0)
New seizure, n (%)	0	2 (3.6)	0	3 (3.1)	0
Hematoma, n (%)	0	1 (1.8)	1 (4.8)	2 (3.1)	1 (4.8)
Reoperation due to complication, n (%)	2 (3.7)	3 (5.4)	1 (4.8)	7 (10.9)	1 (4.8)
Infection, n (%)	1 (1.9)	5 (8.9)	1 (4.8)	10 (15.6)	1 (4.8)
VTE, n/N (%)	0	1 (1.8)	0	5 (7.8)	0
Planned oncological treatment, n (%) Missing, n = 7	11 (20.8)	5 (9.3)	19 (95.0)	36 (58.1)	3 (15.0)
30-day mortality, n (%)	0	1 (1.8)	0	2 (3.1)	0
1-Year mortality, n (%)	3 (5.6)	4 (7.4)	3 (14.3)	9 (14.1)	0

### Pilocytic astrocytoma

Mean age was 36.2 ± 16.9 years and 26 (46.4%) were female. The primary location was in the cerebellum (37.5%) and focal deficit was a common presenting symptom (50.9%) together with ICP related signs and symptoms (58.5%). Nonetheless, most patients had no restriction on the activities of daily life (WHO performance status 0, 51.8%). Gross total resection was achieved in 55.4% of patients and new deficits occurred in 19.6% after surgery. Three patients (5.4%) had a reoperation due to complication. There was one death (1.8%) within 30 days.

### Primitive neuroectodermal tumor

Mean age was 37.0 ± 19.1 years and seven (33.3%) were female. The primary location was in the cerebellum (71.4%) with focal deficits present in most patients (71.4%). Most patients had some restriction on the activities of daily life (WHO performance status I and II, 30.0% each). Gross total resection was achieved in 57.1% of patients and new deficits arose in 33.3% after surgery. There was one (4.8%) reoperation due to complication. There were no deaths within 30 days.

### Ependymoma

Mean age was 51.7 ± 16.3 years and 32 (50.0%) were female. The primary location was in the brain stem/4th ventricle (56.3%) with focal deficits in 42.6% of patients (71.4%). Approximately two thirds of patients had no or limited restriction on the activities of daily life (WHO performance status 0 and I, 36.1% and 32.8%). Gross total resection was achieved in 60.9% of patients and new deficits occurred in 34.4% after surgery. Seven patients (10.9%) underwent a reoperation due to complication. There were two deaths (3.1%) within 30 days.

### Subependymoma

Mean age was 49.8 ± 14.3 years and 4 (19.0%) were female. The primary location was in the brain stem/4^th^ ventricle (52.4%) with focal deficits in 40.0% of the patients. Most patients had no or limited restriction on the activities of daily life (WHO performance status 0 and I, 40.0% and 50.0%). Gross total resection was achieved in 90.5% of patients and new deficits occurred in 19.0% after surgery. There was one patient (4.8%) that underwent a reoperation due to complication. There were no deaths within 30 days of operation.

### Validation in the SEER database

Five thousand, seven hundred, and sixty-nine patients with rare brain tumors were identified in SEER (Table 4). The mean age at diagnosis was 36.4 (GGL), 35.2 (PA), 34.5 (PNET), 46.1 (EP), and 53.3 (SUBEP). While comparable gender distribution was noted for EP, GGL and PA, there was a predominantly male population in Medulloblastoma/PNET and SUBEP. Tumor size at the time of diagnosis was < 4 cm for GGL, PA, EP and SUBEP, while it was 4–6 cm for PNET. Tumors were predominantly unilateral at the time of diagnosis. Almost all (97.4%) medulloblastoma/PNETs and 70.7% of EP were WHO Grade IV and II at the time of diagnosis, respectively, while the majority of SUBEP, GGL and PAs were Grade 1. EP/SUBEP were mainly located in the brain stem and ventricular region; PNET/PA in the cerebellum and GGL in the temporal region. Majority of the cases in each tumor entity underwent gross total resection.

**Table 4:** SEER patients baseline characteristics and resection status

	GGL (n = 680)	PA (n = 1617)	Medulloblastoma/PNET (n = 1285)	EP (n = 1574)	SUBEP (n = 613)
Age at diagnosis, mean (SD)	36.4 (15.3)	35.2 (28.4)	34.5 (30.2)	46.1 (17.1)	53.3 (14.4)
Female, n (%)	323 (47.5)	798 (49.4)	538 (41.9)	732 (46.5)	171 (27.9)
Tumor size, n (%)					
< 4 cm	356 (75.3)	660 (65.2)	332 (42.5)	477 (56.1)	406 (84.4)
4–6 cm	71 (15.0)	296 (29.2)	394 (50.5)	292 (34.3)	59 (12.3)
> 6 cm	46 (9.7)	57 (5.6)	55 (7.0)	82 (9.6)	16 (3.3)
Laterality, n (%)					
Right	269 (51.0)	219 (51.8)	127 (48.7)	134 (49.1)	74 (49.3)
Left	253 (48.0)	203 (48.0)	126 (48.3)	137 (50.2)	72 (48.0)
Bilateral	5 (1.0)	1 (0.3)	8 (3.1)	2 (0.7)	4 (2.7)
Location, n (%)					
Frontal	114 (17.5)	145 (9.7)	76 (6.0)	91 (5.9)	22 (3.6)
Temporal	270 (41.5)	146 (9.8)	26 (2.1)	72 (4.7)	4 (0.7)
Parietal	70 (10.8)	71 (4.7)	35 (2.8)	93 (6.1)	4 (0.7)
Occipital	33 (5.1)	34 (2.3)	11 (0.9)	36 (2.4)	2 (0.3)
Ventricle	31 (4.8)	162 (10.8)	27 (2.1)	361 (23.5)	323 (53.1)
Cerebellum	44 (6.8)	515 (34.4)	960 (76.0)	144 (9.4)	20 (3.3)
Brain stem	21 (3.2)	199 (13.3)	38 (3.0)	472 (30.8)	184 (30.3)
Overlapping lesions	33 (5.1)	64 (4.3)	34 (2.7)	63 (4.1)	2 (0.3)
Brain NOS	34 (5.2)	162 (10.8)	56 (4.4)	203 (13.2)	47 (7.7)
WHO, n (%)					
I	376 (83.7)	663 (94.0)	1 (0.3)	23 (3.7)	293 (93.9)
II	43 (9.6)	22 (3.1)	1 (0.3)	443 (70.7)	18 (5.8)
III	27 (6.0)	13 (1.8)	8 (2.1)	145 (23.1)	1 (0.3)
IV	3 (0.7)	7 (1.0)	379 (97.4)	16 (2.6)	0 (0)
Extent of resection, n (%)					
STR	117 (27.2)	317 (37.2)	219 (32.7)	296 (38.9)	84 (29.0)
GTR	314 (72.9)	536 (62.8)	451 (67.3)	466 (61.2)	206 (71.0)

### Overall survival SBTR and SEER

**Fig. 1:**
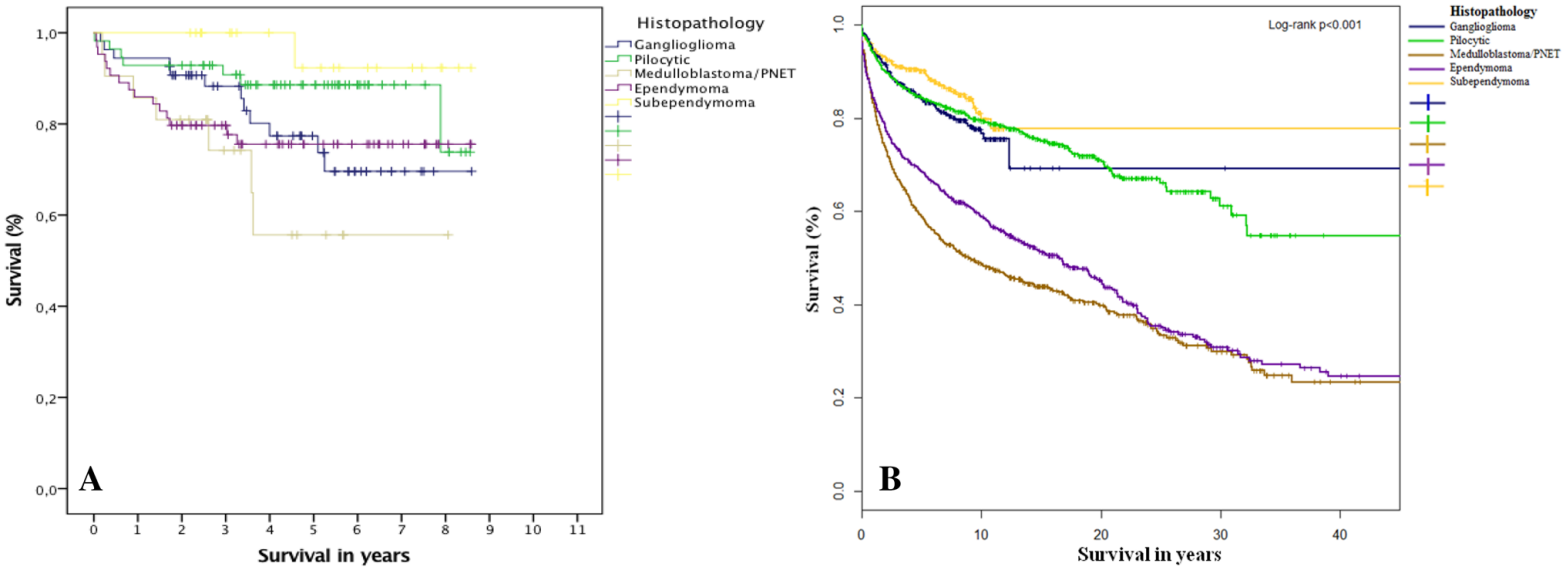
SBTR patients’ overall survival (%) in years (**a**) and SEER patients’ overall survival (%) in years (**b**)

Both SBTR and SEER databases demonstrate SUBEP to have the best overall survival, while Medulloblastoma/PNETs have the worst survival (Fig. 1a and b).

## Discussion

In this nationwide registry-based study spanning from 2009–2015, we describe the baseline, tumor and outcome characteristics as well as the 30-day complication rate after intracranial surgery for rare brain tumors in the adult population. To the best of our knowledge, we are the first to systematically report on short-term surgical outcome in terms of morbidity and mortality in adults with rare brain tumors. We provide separate data per entity, and there are variations in short-term outcome among the different tumor entities, commented in detail in subsections below.

### Ganglioglioma

The adult literature is scarce, but some data from mixed adult/pediatric cohorts can be used for comparison, although surgical series mainly focus on seizure outcome [5–8]. Epilepsy is present in 28–85% of patients [4, 5, 10, 11], with the temporal lobe location in up to 62% [5]. Among the adults in our study 64.2% had seizures and 35% of GGL were located in the temporal lobe. Like the literature, we report GTR in > 60% [4, 5, 10], and GTR has been demonstrated to result in better seizure control/freedom than STR or biopsy only [4]. Short term surgical morbidity and mortality is seldom addressed. There are only two recent reports available, demonstrating 1–17% complication/morbidity frequency [12, 13] and another reporting of < 3% mortality [5]. The wide span between the reported complication/morbidity could be caused by the difference in the definition of a complication. In our cohort, new deficits after surgery were reported in 11.1% of cases, with 3.7% in need of re-operation due to complication(s). Finally, whereas adjuvant oncological treatment was planned in 20.8% of our cohort, there is little evidence of adjuvant treatment in other reports, while some even report sporadic or no adjuvant treatment being offered [5, 11], and while the reason for this can only be speculated upon, it could be related to the fact that 11.1% of gangliogliomas in our cohort were anaplastic, while the WHO degree was not disclosed in the other reports, with these possibly comprising only benign (WHO grade 1) entities.

### Pilocytic astrocytoma

Treatment outcomes in adult PA has been reported on previously [14–18], albeit mainly with focus on the overall treatment outcome and risk of recurrence rather than surgical morbidity and mortality. Overall, as in our cohort, previous reports describe the mean age of adult pilocytic astrocytoma diagnosis to be in the late 20 s—early 30 s, with localization primarily in the cerebellum and/or brainstem [14–18], with signs and symptoms of raised ICP due to the development of hydrocephalus present in up to 90% in certain reports [16]. Gross total resection has been achieved in approximately 50% of cases in the most modern series [14], comparable to our findings. However, the reported 7% permanent postoperative deficits are markedly lower than in our cohort (19.2%), however there is a difference in reporting where Kamila et al.only reported on permanent deficits [14]. Of note, earlier series by Ye et al*.* [15] reported up to 20% morbidity, 35% complication frequency and a 10% mortality, which are all higher than numbers reported in our cohort and a possible indication of improvement in surgical care over time. Finally, varying degree of adjuvant treatment has been reported, from 6–22% [14, 15, 17] with our frequency being 9.3%, which is more in line with overall recommendations from recent literature only to use radiotherapy as salvage [19], further substantiated by the demonstration of prolonged PFS but equal OS between adult patients with pilocytic astrocytoma that underwent GTR with- or without adjuvant radiotherapy [20].

### Primitive neuroectodermal tumor

PNET stands for primitive neuroectodermal tumor, among which medulloblastoma is the prototype (as defined by the 2007 WHO criteria used in the current SBTR analyses), and as such constitutes most patients reported on [21–23]. Reports on outcome in adult medulloblastoma are rare, and in the few that exist, limited or no attention has been given to demographic and surgical characteristics, while focusing on the overall treatment outcome [22, 24–29], making comparison difficult.

Those that report on PNET in adults, report a peak incidence in the late 20 s [29], but again, this is highly dependent of whether the patients were considered adults as > 15 y/o or > 18 y/o. In our cohort, the mean age at diagnosis was the late 30 s, consistent with the patients in our cohort included > 18 y/o. The literature describes an up to 20% incidence of metastatic/multifocal disease [27] upon diagnosis, with signs of increased ICP present in the majority of patients [22, 24, 27], similarly to our results with multifocal disease in 23.8% cases and 81% having signs of increased ICP. This correlates well to the severely affected (in comparison to the other rare entities) performance status with 30% of the patients in WHO class I and II, respectively, but even 15% in WHO class IV. In 57.1% GTR was achieved, a frequency that is both lower [21] and higher [27] in literature. Comparing surgical morbidity with the scarce literature is difficult due to non-uniform reporting. We report that 33% of patients experience deficit within 30 days, others have found morbidity in 19% of cases [25]. Further, being a malignant disease with a high symptom burden, there is an indication for timely intervention (all cases operated within < 1 month after radiological diagnosis) as is the need for adjuvant therapy. In the literature almost all reported cases receive adjuvant oncological therapy [21, 22, 28, 29], as was the case in our cohort.

### Ependymoma

There are several publications on outcome in adult patients with intracranial ependymomas. The demographic characteristics match these of our cohort; with the patients diagnosed mainly in their late 40 s—early 50 s, presenting with signs/symptoms of raised intracranial pressure, with most tumors located infratentorial and of WHO grade 2 histology [30–35]. GTR is reported in 58–74% of cases [34, 35], comparable to our result. Concerning surgical safety profile, as is the case with the other rare entities, the previous reports focus mainly on overall treatment outcome. However, patients with ependymoma in our material experience a high burden of neurological morbidity, but results are comparable to others (27–59%) even though the higher frequency is reported from a mixed intracranial/spinal series [30, 35]. The rather high frequency illustrates that this entity is often located in a sensitive area (56.3% brainstem/4th ventricle location in our cohort) and is approached aggressively in terms of desired GTR. Further, although not previously reported by others, we even report a re-operation frequency of 10.9% and a postoperative infection rate of 15.6%, which is high but comparable to the 11% reported by Acquage et al*.* [30]. Finally, our perioperative mortality is 3.1%, comparable to 4.7–7.9% reported by others [32, 34].

### Subependymoma

SUBEP is a benign (WHO grade 1) tumor, mostly located in the ventricles of the brain, as is the case in our cohort (81% located primarily in one of the four ventricles), with middle aged male preponderance (81% in our cohort) [36–41]. There are several recent publications addressing the surgical outcome of SUBEP, albeit mostly small case series, reporting on patients often presenting with headache (> 60%) due to the predominantly ventricular location of these tumors, with risk of secondary hydrocephalus [40, 41]. GTR is usually reported to achieved in > 70% of surgeries [40]. Surgical morbidity of 18–33% [39–41] with new focal deficits addressed only by Bi Z et al*.* as present in 14% of cases [39]. Further, even though mortality is seldom seen, Bi Z et al*.* reports a 2.3% perioperative mortality [39]. In general, these numbers are in line with the reported frequencies in our cohort, although the frequency of new deficits after surgery is somewhat higher (19%) – which could be associated with > 90% GTR as a sign of more aggressive tumor removal.

### Comparison of SEER and SBTR results

While the total patients included from the SBTR is only 216 as compared to 5,769 from the SEER database, the SBTR data provide novel and useful information concerning short-term surgical outcome data in a modern series—data which are not available in the SEER database. Comparing the datasets available in both SEER and SBTR we obtain a gross idea of the clinical profiles of the rare brain tumor patients, and to a large extent the SEER database validates the SBTR. This validation demonstrate that patient and tumor characteristics seem generalizable to other settings. The strength of the SBTR is the national coverage, but also that more clinical and outcome characteristics are available compared to SEER, while the SEER database has a much larger data set and longer follow-up time. We believe that in addition to the baseline characteristics of the studied population, the operative and outcome results can be used as a benchmark for further studies and in improving patient care.

### Strengths and limitations

Limitations of this study include those typical of register-based studies with limited details and without possibility to complete missing data since data is provided without identification from the registry holder. Further, there is a lack of detail for variables and long-term follow-up, for instance if the neurological deficit is transient or permanent, as well as considerable possibilities for interpretation of certain variables such as postoperative hematoma.

Strengths include population-based inclusion of a rather large number of patients with rare brain tumors from a recent time period where data is reported in a continuous, prospective and standardized fashion. SEER database has also been analyzed along with the SBTR and found to a large degree to corroborate the SBTR dataset, which increase the external validity our results.

## Conclusion

In this nation-wide registry based-study, we benchmark the current symptomatology, tumor characteristics and outcome after intracranial surgery for rare brain tumors—ganglioglioma, pilocytic astrocytoma, primitive neuroectodermal tumor/medulloblastoma, ependymoma and subependymoma—in adult patients in Sweden. This is the largest study focusing on surgical effectiveness and safety when treating rare brain tumors in adult patients, with data from the SBTR to a large extent comparable to those reported in the SEER, increasing the external validity of our results. We believe this information to be of value for both caregivers and patients as to what to expect being operated on due to a rare brain tumor entity.
